# Adenoid Cystic Carcinoma of the Breast: A Case Report

**DOI:** 10.7759/cureus.25131

**Published:** 2022-05-19

**Authors:** Nicole M Reyes, Leva Gorji, Grant Huish, Andrew Archer

**Affiliations:** 1 Department of Surgery, Kettering Health Network, Dayton, USA

**Keywords:** breast surgery, breast tumor, breast cancer, adenoid cystic carcinoma, rare

## Abstract

Adenoid cystic carcinoma (ACC), characterized by proliferating glands and stromal and basement membrane elements, is a tumor most commonly occurring in the salivary glands; very rarely does this tumor present as a primary tumor in the breast. We present the case of a 53-year-old female who presented with a breast mass noted to be concerning on imaging and confirmed to be ACC on biopsy. The mass was amenable to surgical excision with referrals to medical and radiation oncology. Due to the rarity of the disease, there is no consensus regarding the optimal treatment strategy for the pathology, including the use of chemotherapy and radiation. Long-term follow-up is, however, recommended in order to monitor for recurrence.

## Introduction

We present the case of a 53-year-old female who presented with a right-sided breast mass, noted to be adenoid cystic carcinoma (ACC) on core needle biopsy with subsequent confirmation of pathology on formal, surgical excision. ACC, characterized by proliferating glands and stromal and basement membrane elements, is a tumor most commonly occurring in the salivary glands; however, it may also occur in the nasopharynx, bronchopulmonary tree, uterus, cervix, and kidneys. Very rarely, ACC presents as a breast neoplasm, comprising 0.1-1% of all breast tumors. ACC neoplasm of the breast has been exclusively associated with the female gender and commonly estrogen (ER), progesterone (PR), and HER2/neu negative on immunochemistry. Triple-negative breast cancer (TNBC) accounts for a minority of breast cancers and is typically associated with a poor prognosis. However, primary ACC breast cancers do possess a favorable outcome despite being TNBC [1-2].

## Case presentation

**Figure 1:**
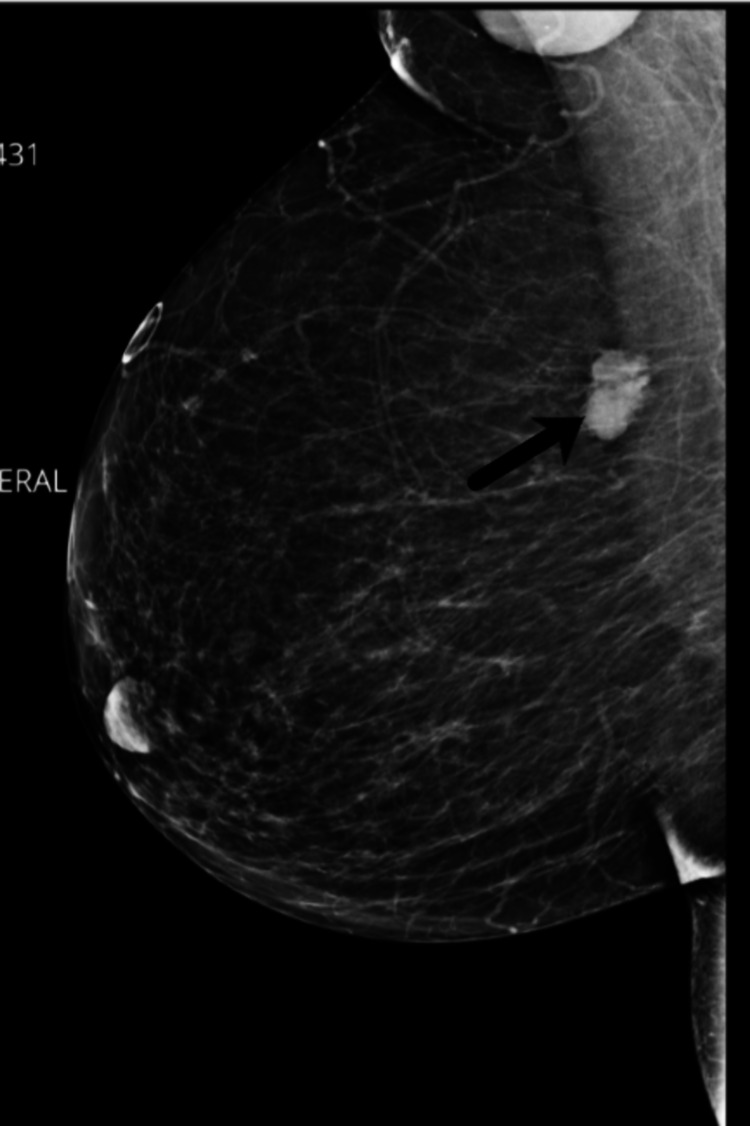
Mammogram with the concerning lesion Black arrow indicating the lesion of consideration - a 2 cm, hyperdense mass with indistinct margins at approximately the 9 o’clock position of the right breast

**Figure 2:**
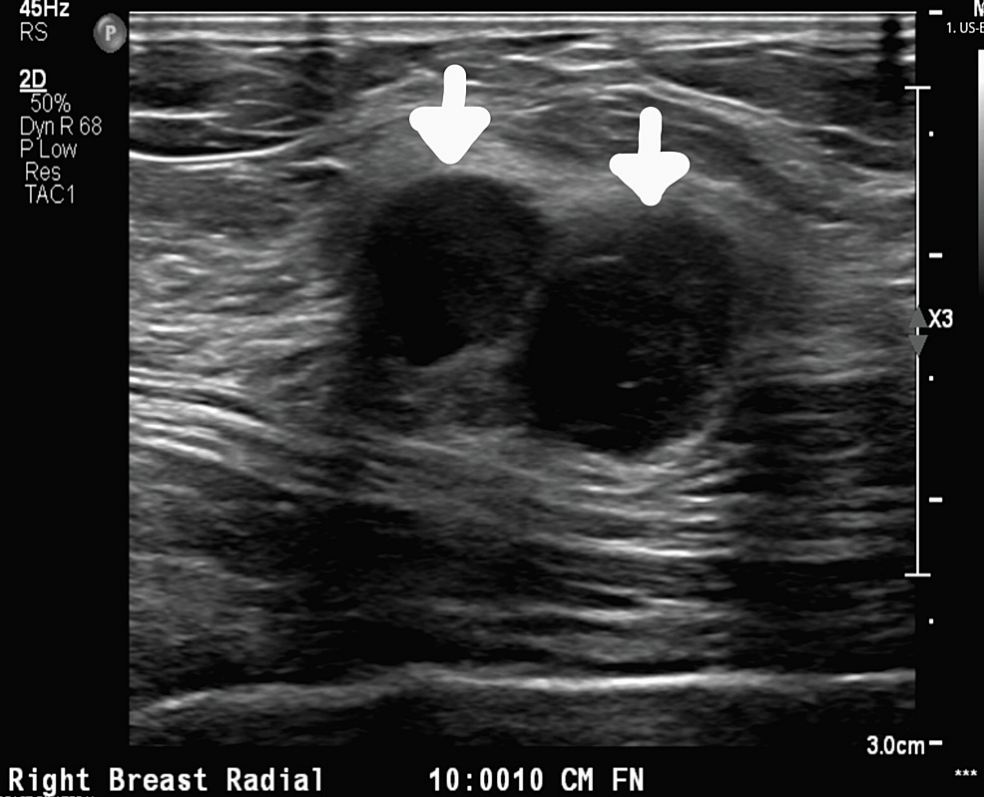
Follow-up ultrasound A bilobed 2x1.6 cm mass at the 10 o’clock position 10 cm from the nipple

**Figure 3:**
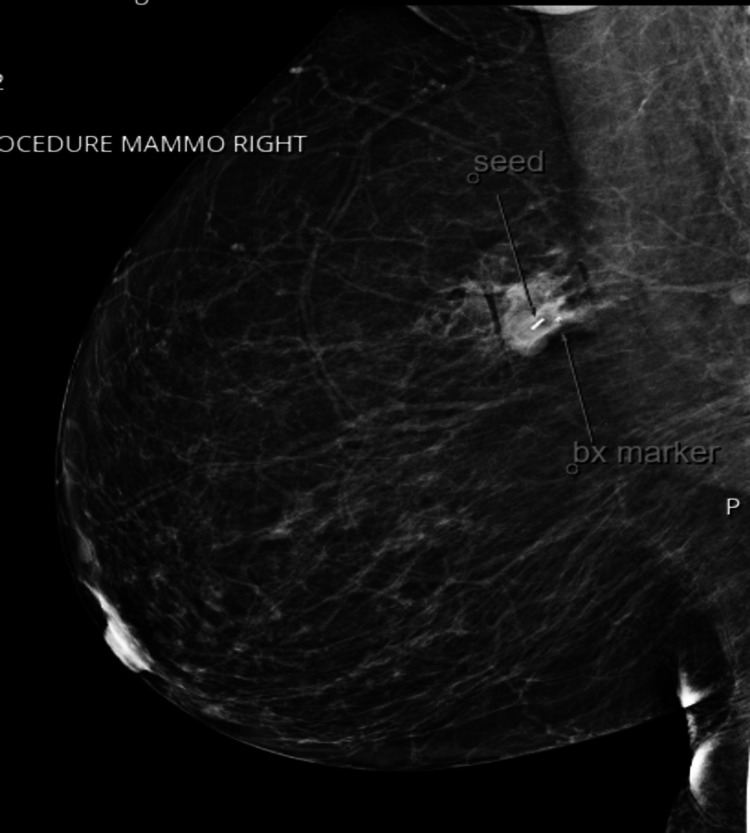
Post-procedure mammogram after insertion of seed

We present the case of a 53-year-old female patient with poor compliance with a primary care provider who presented with a right-sided breast mass. The patient’s family history was pertinent for a mother with breast cancer; the patient did not know the type of breast cancer. The patient’s surgical history was remarkable for a c-section and tonsillectomy. The patient re-established with a primary care provider after 10 years. Upon initial workup, the patient was noted to have a thyroid nodule and Hashimoto's disease. On screening mammography, the patient was also noted to have an oval, 2 cm, hyperdense mass with indistinct margins at approximately the 9 o’clock position of the right breast (Figure 1). This was determined to be a Breast Imaging Reporting and Database System (BI-RADS) score of 0, warranting further imaging evaluation. A follow-up 3D tomosynthesis and breast ultrasound revealed a bilobed 2x1.6 cm mass at the 10 o’clock position 10 cm from the nipple (Figure 2), categorized as BI-RADS 5. A concordant ultrasound-guided biopsy was obtained, which was ER/PR (-) and HER-2 (-) on immunohistochemistry (IHC) and fluorescence in situ hybridization (FISH) with a Ki-67 of 20%. IHC stains of the specimen were performed for further analysis with evidence of adenoid cystic carcinoma - the specimen was CD117 positive, SMMS focally positive with P63, P40 highlighting myoepithelial cells, EMA and CK7 highlighting a ductal component, and E-cadherin and P120 highlighting membranous staining. A surgical consult was obtained with plans for wide excision of right breast cancer with seed localization and a right-sided sentinel lymph node biopsy. Ultrasound-guided injection of a radioactive I-125 seed was placed (Figure 3) prior to the surgical intervention in the previously biopsied mass site. A dose rate meter device was utilized in order to confirm seed positioning within the breast. Radiotracer and methylene blue were injected into the breast in order to appropriately identify the sentinel lymph nodes intraoperatively. A right-sided partial mastectomy with seed localization was performed with a right sentinel lymph node biopsy. Intraoperative radiography of the specimen did confirm the presence of the radioactive seen and the prior biopsy clip. Final pathology revealed a 2.2 x 2.0 x 1.6 cm mass revealing an adenoid cystic carcinoma, Grade 1, negative margins, with the closest margin being 1.0 mm at the inferior aspect and one sentinel lymph, which was benign, making the final pathological stage pT2, N0. The patient has been seen postoperatively, is healing appropriately, and has been referred to medical and radiation oncology for further opinion regarding adjuvant therapy. However, due to the patient's poor compliance, she has not followed up for further evaluation.

## Discussion

Primary ACC of the breast accounts for 12% of ACC neoplasms. As this is an extremely rare primary malignant tumor of the breast, a unique set of identifiable physical exam or imaging characteristics have not been developed. There are two subtypes of ACC that have been described in the literature: a cribriform/tubular histological subtype in which tumor cells are predominantly cribriform and tubular and a solid histological subtype in which tumor cells are predominantly solid tumor nests with cord patterns [1]. Because the solid histological subtype carries a higher risk of malignancy, they have been further classified into three grades when present in the salivary glands: Grade I has no solid component, Grade II has <30% solid component, and Grade III >30% solid component [1-5]. Our patient’s tumor was the tubular subtype; furthermore, the tumor was also identified to be triple-negative on immunohistochemical analysis, consistent with the majority of breast ACC [5-8]. These tumors exhibit immunoreactivity for p63 and CD17. While ACC is a slow-growing tumor, it does exhibit lymphatic spread; therefore, a sentinel lymph node biopsy is an appropriate indicated approach. The patient is triple-negative, therefore, adjuvant therapy with chemotherapy may be discussed on an individualized basis based on comorbidities, size of the tumor, triple-negative status, and subtype of ACC [8].

While ACC of the head and neck is typically treated with surgical excision and supplemental center-specific guidelines regarding adjuvant radiation, current recommendations regarding treatment of ACC of the breast are extremely limited [9]. A similar regimen of breast conservation therapy with postoperative radiation was advised to our patient. Previous case reports pertaining to ACC of the breast all indicate triple-negative disease, thereby not requiring hormonal therapy; however, the role of systemic chemotherapy becomes questionable as the breast tumor does qualify for the treatment given the triple-negative status but has exhibited excellent prognosis with breast conservation therapy and radiation [10]. ACC of the head and neck exhibits frequent local recurrence, occasional metastases to the lymph nodes, and rare incidence of distant metastasis; however, the prognosis is better and metastasis is lower when comparing ACC of the breast to other regions [10-15]. Due to the delayed risk of recurrence, appropriate surveillance and follow-up are imperative for the long management of the tumor. Given the role and uptake of the fluorodeoxyglucose (FDG)-positron emission tomography (PET) scan has not been formally established in head and neck ACC, it was not pursued for our patient [14-15].

The role of postoperative radiation therapy is also unclear after wide excision of ACC of the breast. Recent guidelines have shown that clinicians can consider omitting postoperative breast radiation after breast-conserving surgery in invasive breast carcinomas with favorable tumor characteristics if the patient is aged 70 years or older [16]. It appears, for now, that administering postoperative breast radiation for ACC of the breast will need to be determined on a case-by-case basis.

## Conclusions

Due to the rare nature of this breast cancer, limited data are available regarding optimal treatment and management. While triple-negative breast cancers are typically high-grade tumors with some requiring systemic therapy, ACC of the breast does appear to have a positive prognosis with systemic therapy providing negligible benefit to survival outcomes. Therefore, further studies and continued recognition of the disease process are required in order to establish the optimal treatment algorithm for the disease.
